# Supplementary material for: Development of a Novel RT-qPCR Detecting Method of Covert Mortality Nodavirus (CMNV) for the National Proficiency Test in Molecular Detection
Source: Viruses. 2022 Jul 5;14(7):1475. doi: 10.3390/v14071475 (PMC9319686; doi:10.3390/v14071475)
Supplement: Supplementary file 1 [file viruses-14-01475-s001.zip › viruses-1724135-supplementary.pdf]

Supplemental Table S1. The clinical sample information, and the comparison of detection results of the newly developed TaqMan RT-qPCR and Li′ s (2018) TaqMan RT-qPCR.

number	Sample number	Sample type	Sample source	Sample symptom	The Taq Man RT-qPCR	Li's (2018) Taq Man RT-qPCR
1	20210320001	*P. vannamei*	Shandong province	/	- - -	-
2	20210320002	*P. vannamei*	Shandong province	shell softening, muscle whitening and necrosis at the acute stage	+ + +	+
3	20210320003	*P. vannamei*	Shandong province	/	- - -	-
4	20210320004	*P. vannamei*	Shandong province	/	- - -	-
5	20210320005	*P. vannamei*	Shandong province	/	- - -	-
6	20210320006	*P. vannamei*	Shandong province	shell softening, muscle whitening and necrosis at the acute stage	+ + +	+
7	20210320007	*P. vannamei*	Shandong province	/	- - -	-
8	20210320008	*P. vannamei*	Shandong province	shell softening, muscle whitening and necrosis at the acute stage	+ + +	+
9	20210320009	*P. vannamei*	Shandong province	shell softening, muscle whitening and necrosis at the acute stage	+ + +	+
10	20210320010	*P. vannamei*	Shandong province	shell softening, muscle whitening and necrosis at the acute stage	+ + +	+
11	20210320011	*P. vannamei*	Shandong province	shell softening, muscle whitening and necrosis at the acute stage	+ + +	+
12	20210320012	*P. vannamei*	Shandong province	shell softening, muscle whitening and necrosis at the acute stage	+ + +	+
13	20210320013	*P. vannamei*	Shandong province	/	- - -	-
14	20210320014	*P. vannamei*	Shandong province	/	- - -	-
15	20210320015	*P. vannamei*	Shandong province	/	- - -	-
16	20210320016	*P. vannamei*	Shandong province	/	- - -	-
17	20210320017	*P. vannamei*	Shandong province	/	- - -	-
18	20210320018	*P. vannamei*	Shandong province	/	- - -	-
19	20210320019	*P. vannamei*	Shandong province	shell softening, muscle whitening and necrosis at the acute stage	+ + +	+
20	20210320020	*P. vannamei*	Shandong province	/	- - -	-
21	20210320021	*P. vannamei*	Shandong province	shell softening, muscle whitening and necrosis at the acute stage	+ + +	+
22	20210320022	*P. vannamei*	Shandong province	/	- - -	-
23	20210320023	*P. vannamei*	Shandong province	/	- - -	-
24	20210320024	*P. vannamei*	Shandong province	/	- - -	-
25	20210320025	*P. vannamei*	Shandong province	/	- - -	-
26	20210320026	*P. vannamei*	Shandong province	shell softening, muscle whitening and necrosis at the acute stage	+ + +	+
27	20210320027	*P. vannamei*	Shandong province	shell softening, muscle whitening and necrosis at the acute stage	+ + +	+
28	20210320028	*P. vannamei*	Shandong province	shell softening, muscle whitening and necrosis at the acute stage	+ + +	+
29	20210320029	*P. vannamei*	Shandong province	/	- - -	-
30	20210320030	*P. vannamei*	Shandong province	/	- - -	-
31	20210320031	*P. vannamei*	Shandong province	/	- - -	-
32	20210320032	*P. vannamei*	Shandong province	shell softening, muscle whitening and necrosis at the acute stage	+ + +	+
33	20210320033	*P. vannamei*	Shandong province	shell softening, muscle whitening and necrosis at the acute stage	+ + +	+
34	20210320034	*P. vannamei*	Shandong province	/	- - -	-
35	20210320035	*P. vannamei*	Shandong province	shell softening, muscle whitening and necrosis at the acute stage	+ + +	+
36	20210320036	*P. vannamei*	Shandong province	/	- - -	-
37	20210320037	*P. vannamei*	Shandong province	/	- - -	-
38	20210320038	*P. vannamei*	Shandong province	/	- - -	-
39	20210320039	*P. vannamei*	Shandong province	shell softening, muscle whitening and necrosis at the acute stage	+ + +	+
40	20210320040	*P. vannamei*	Shandong province	shell softening, muscle whitening and necrosis at the acute stage	+ + +	+
41	20210320041	*P. vannamei*	Shandong province	shell softening, muscle whitening and necrosis at the acute stage	+ + +	+
42	20210320042	*P. vannamei*	Shandong province	/	- - -	-
43	20210320043	*P. vannamei*	Shandong province	/	- - -	-

44	20210320044	*P. vannamei*	Shandong province	/	-	-	-	-
45	20210320045	*P. vannamei*	Shandong province	/	-	-	-	-
46	20210320046	*P. vannamei*	Shandong province	/	-	-	-	-
47	20210320047	*P. vannamei*	Shandong province	/	-	-	-	-
48	20210320048	*P. vannamei*	Shandong province	shell softening, muscle whitening and necrosis at the acute stage	+	+	+	+
49	20210320049	*P. vannamei*	Shandong province	/	-	-	-	-
50	20210320050	*P. vannamei*	Shandong province	/	-	-	-	-
51	20210320051	*P. vannamei*	Shandong province	/	-	-	-	-
52	20210320052	*P. vannamei*	Shandong province	/	-	-	-	-
53	20210320053	*P. vannamei*	Shandong province	/	-	-	-	-
54	20210320054	*P. vannamei*	Shandong province	/	-	-	-	-
55	20210320055	*P. vannamei*	Shandong province	shell softening, muscle whitening and necrosis at the acute stage	+	+	+	+
56	20210320056	*P. vannamei*	Shandong province	shell softening, muscle whitening and necrosis at the acute stage	+	+	+	+
57	20210320057	*P. vannamei*	Shandong province	/	-	-	-	-
58	20210320058	*P. vannamei*	Shandong province	/	-	-	-	-
59	20210320059	*P. vannamei*	Shandong province	/	-	-	-	-
60	20210320060	*P. vannamei*	Shandong province	/	-	-	-	-
61	20210320061	*P. vannamei*	Shandong province	/	-	-	-	-
62	20210320062	*P. vannamei*	Shandong province	shell softening, muscle whitening and necrosis at the acute stage	+	+	+	+
63	20210320063	*P. vannamei*	Shandong province	/	-	-	-	-
64	20210320064	*P. vannamei*	Shandong province	/	-	-	-	-
65	20210320065	*P. vannamei*	Shandong province	/	-	-	-	-
66	20210320066	*P. vannamei*	Shandong province	/	-	-	-	-
67	20210320067	*P. vannamei*	Shandong province	/	-	-	-	-
68	20210320068	*P. vannamei*	Shandong province	shell softening, muscle whitening and necrosis at the acute stage	+	+	+	+
69	20210320069	*P. vannamei*	Shandong province	shell softening, muscle whitening and necrosis at the acute stage	+	+	+	+
70	20210320070	*P. vannamei*	Shandong province	shell softening, muscle whitening and necrosis at the acute stage	+	+	+	+
71	20210320071	*P. vannamei*	Shandong province	shell softening, muscle whitening and necrosis at the acute stage	+	+	+	+
72	20210320072	*P. vannamei*	Shandong province	/	-	-	-	-
73	20210320073	*P. vannamei*	Shandong province	/	-	-	-	-
74	20210320074	*P. vannamei*	Shandong province	/	-	-	-	-
75	20210320075	*P. vannamei*	Shandong province	/	-	-	-	-
76	20210320076	*P. vannamei*	Shandong province	/	-	-	-	-
77	20210320077	*P. vannamei*	Shandong province	shell softening, muscle whitening and necrosis at the acute stage	+	+	+	+
78	20210320078	*P. vannamei*	Shandong province	/	-	-	-	-
79	20210320079	*P. vannamei*	Shandong province	/	-	-	-	-
80	20210320080	*P. vannamei*	Shandong province	/	-	-	-	-
81	20210320081	*P. vannamei*	Shandong province	/	-	-	-	-
82	20210320082	*P. vannamei*	Shandong province	/	-	-	-	-
83	20210320083	*P. vannamei*	Shandong province	/	-	-	-	-
84	20210320084	*P. vannamei*	Shandong province	/	-	-	-	-
85	20210320085	*P. vannamei*	Shandong province	/	-	-	-	-
86	20210320086	*P. vannamei*	Shandong province	/	-	-	-	-
87	20210917001	*Macrobrachium rosenbergii*	Jiangsu province	/	-	-	-	-
88	20210917002	*Macrobrachium rosenbergii*	Jiangsu province	/	-	-	-	-
89	20210917003	*Macrobrachium rosenbergii*	Jiangsu province		+	-	-	+
90	20210917004	*Macrobrachium rosenbergii*	Jiangsu province	/	-	-	-	-
91	20210917005	*Macrobrachium rosenbergii*	Jiangsu province	/	-	-	-	-
92	20210917006	*Macrobrachium rosenbergii*	Jiangsu province	shell softening	+	+	+	+
93	20210917007	*Macrobrachium rosenbergii*	Jiangsu province	/	-	-	-	+
94	20210917008	*Macrobrachium rosenbergii*	Jiangsu province	/	-	-	-	-

95	20210917009	*Macrobrachium rosenbergii*	Jiangsu province	/	-	-	-	-
96	20210917010	*Macrobrachium rosenbergii*	Jiangsu province	/	-	-	-	+
97	20210917011	*Macrobrachium rosenbergii*	Jiangsu province	/	-	-	-	-
98	20210917012	*Macrobrachium rosenbergii*	Jiangsu province	/	-	-	-	-
99	20210917013	*Macrobrachium rosenbergii*	Jiangsu province	/	-	-	-	-
100	20210917014	*Macrobrachium rosenbergii*	Jiangsu province	/	-	-	-	-
101	20210917015	*Macrobrachium rosenbergii*	Jiangsu province	/	-	-	-	-
102	20210917016	*Macrobrachium rosenbergii*	Jiangsu province	/	-	-	-	-
103	20210917017	*Macrobrachium rosenbergii*	Jiangsu province	/	-	-	-	-
104	20210917018	*Macrobrachium rosenbergii*	Jiangsu province	/	-	-	-	-
105	20210917019	*Macrobrachium rosenbergii*	Jiangsu province	/	-	-	-	-
106	20210917020	*Macrobrachium rosenbergii*	Jiangsu province	shell softening	+	+	-	+
107	20210917021	*Macrobrachium rosenbergii*	Jiangsu province	/	-	-	-	-
108	20210917022	*Macrobrachium rosenbergii*	Jiangsu province	/	-	-	-	-
109	20210917023	*Macrobrachium rosenbergii*	Jiangsu province	/	-	-	-	-
110	20210917024	*Macrobrachium rosenbergii*	Jiangsu province	/	-	-	-	-
111	20210917025	*Macrobrachium rosenbergii*	Jiangsu province	/	-	-	-	-
112	20210917026	*Macrobrachium rosenbergii*	Jiangsu province	/	-	-	-	-
113	20211228001	*P. vannamei*	Shandong province	/	-	-	-	-
114	20211228002	*P. vannamei*	Shandong province	/	-	-	-	-
115	20211228003	*P. vannamei*	Shandong province	/	-	-	-	-
116	20211228004	*P. vannamei*	Shandong province	/	-	-	-	-
117	20211228005	*P. vannamei*	Shandong province	/	-	-	-	-
118	20211228006	*P. vannamei*	Shandong province	/	-	-	-	-
119	20211228007	*P. vannamei*	Shandong province	/	-	-	-	-
120	20211228008	*P. vannamei*	Shandong province	/	-	-	-	-
121	20211228009	*P. vannamei*	Shandong province	/	-	-	-	-
122	20211228010	*P. vannamei*	Shandong province	/	-	-	-	-
123	20211228011	*P. vannamei*	Shandong province	/	-	-	-	-
124	20211228012	*P. vannamei*	Shandong province	/	-	-	-	-
125	20211228013	*P. vannamei*	Shandong province	/	-	-	-	-
126	20211228014	*P. vannamei*	Shandong province	/	-	-	-	-
127	20211228015	*P. vannamei*	Shandong province	/	-	-	-	-
128	20211228016	*P. vannamei*	Shandong province	/	-	-	-	-
129	20211228017	*P. vannamei*	Shandong province	/	-	-	-	-
130	20211228018	*P. vannamei*	Shandong province	/	-	-	-	-
131	20211228019	*P. vannamei*	Shandong province	/	-	-	-	-
132	20211228020	*P. vannamei*	Shandong province	/	-	-	-	-
133	20211228021	*P. vannamei*	Shandong province	/	-	-	-	-
134	20211228022	*P. vannamei*	Shandong province	/	-	-	-	-
135	20211228023	*P. vannamei*	Shandong province	/	-	-	-	-
136	20211228024	*P. vannamei*	Shandong province	/	-	-	-	-
137	20211228025	*P. vannamei*	Shandong province	/	-	-	-	-
138	20211228026	*P. vannamei*	Shandong province	/	-	-	-	-
139	20211228027	*P. vannamei*	Shandong province	/	-	-	-	-
140	20211228028	*P. vannamei*	Shandong province	/	-	-	-	-
141	20211228029	*P. vannamei*	Shandong province	/	-	-	-	-
142	20211228030	*P. vannamei*	Shandong province	/	-	-	-	-
143	20211228031	*P. vannamei*	Shandong province	/	-	-	-	-
144	20211228032	*P. vannamei*	Shandong province	/	-	-	-	-
145	20211228033	*P. vannamei*	Shandong province	/	-	-	-	-

146	20211228034	*P. vannamei*	Shandong province	/	-	-	-	-
147	20211228035	*P. vannamei*	Shandong province	/	-	-	-	-
148	20211228036	*P. vannamei*	Shandong province	/	-	-	-	-
149	20211228037	*P. vannamei*	Shandong province	/	-	-	-	-
150	20211228038	*P. vannamei*	Shandong province	/	-	-	-	-
151	20211228039	*P. vannamei*	Shandong province	empty stomach and guts, shell softening, muscle whitening and necrosis at the acute stage	+	+	+	+
152	20211228040	*P. vannamei*	Shandong province	empty stomach and guts, shell softening, muscle whitening and necrosis at the acute stage	+	+	+	+
153	20211228041	*P. vannamei*	Shandong province	empty stomach and guts, shell softening, muscle whitening and necrosis at the acute stage	+	+	+	+
154	20211228042	*P. vannamei*	Shandong province	empty stomach and guts, shell softening, muscle whitening and necrosis at the acute stage	+	+	+	+
155	20211228043	*P. vannamei*	Shandong province	empty stomach and guts, shell softening, muscle whitening and necrosis at the acute stage	+	+	+	+
156	20211228044	*P. vannamei*	Shandong province	empty stomach and guts, shell softening, muscle whitening and necrosis at the acute stage	+	+	+	+
157	20211228045	*P. vannamei*	Shandong province	empty stomach and guts, shell softening, muscle whitening and necrosis at the acute stage	+	+	-	+
158	20211228046	*P. vannamei*	Shandong province	empty stomach and guts, shell softening, muscle whitening and necrosis at the acute stage	+	+	+	+
159	20211228047	*P. vannamei*	Shandong province	empty stomach and guts, shell softening, muscle whitening and necrosis at the acute stage	+	+	+	+
160	20211228048	*P. vannamei*	Shandong province	empty stomach and guts, shell softening, muscle whitening and necrosis at the acute stage	+	+	+	+
161	20211228049	*P. vannamei*	Shandong province	empty stomach and guts, shell softening, muscle whitening and necrosis at the acute stage	+	+	+	+
162	20211228050	*P. vannamei*	Shandong province	empty stomach and guts, shell softening, muscle whitening and necrosis at the acute stage	+	+	+	+
163	20211228051	*P. vannamei*	Shandong province	empty stomach and guts, shell softening, muscle whitening and necrosis at the acute stage	+	+	+	+
164	20211228052	*P. vannamei*	Shandong province	empty stomach and guts, shell softening, muscle whitening and necrosis at the acute stage	+	+	+	+
165	20211228053	*P. vannamei*	Shandong province	empty stomach and guts, shell softening, muscle whitening and necrosis at the acute stage	+	+	+	+
166	20211228054	*P. vannamei*	Shandong province	empty stomach and guts, shell softening, muscle whitening and necrosis at the acute stage	+	+	+	+
167	20211228055	*P. vannamei*	Shandong province	empty stomach and guts, shell softening, muscle whitening and necrosis at the acute stage	+	+	+	+
168	20211228056	*P. vannamei*	Shandong province	empty stomach and guts, shell softening, muscle whitening and necrosis at the acute stage	+	+	+	+
169	20211228057	*P. vannamei*	Shandong province	empty stomach and guts, shell softening, muscle whitening and necrosis at the acute stage	+	+	+	+
170	20211228058	*P. vannamei*	Shandong province	empty stomach and guts, shell softening, muscle whitening and necrosis at the acute stage	+	+	+	+
171	20211228059	*P. vannamei*	Shandong province	empty stomach and guts, shell softening, muscle whitening and necrosis at the acute stage	+	+	+	+

172	20211228060	*P. vannamei*	Shandong province	empty stomach and guts, shell softening, muscle whitening and necrosis at the acute stage	+	+	+		+
173	20211228061	*P. vannamei*	Shandong province	empty stomach and guts, shell softening, muscle whitening and necrosis at the acute stage	+	+	+		+
174	20211228062	*P. vannamei*	Shandong province	empty stomach and guts, shell softening, muscle whitening and necrosis at the acute stage	+	+	+		+
175	20211228063	*P. vannamei*	Shandong province	empty stomach and guts, shell softening, muscle whitening and necrosis at the acute stage	+	+	+		+
176	20211228064	*P. vannamei*	Shandong province	empty stomach and guts, shell softening, muscle whitening and necrosis at the acute stage	+	+	+		+
177	20211228065	*P. vannamei*	Shandong province	empty stomach and guts, shell softening, muscle whitening and necrosis at the acute stage	+	+	+		+
178	20211228066	*P. vannamei*	Shandong province	empty stomach and guts, shell softening, muscle whitening and necrosis at the acute stage	+	+	+		+
179	20211228067	*P. vannamei*	Shandong province	empty stomach and guts, shell softening, muscle whitening and necrosis at the acute stage	+	+	+		+
180	20211228068	*P. vannamei*	Shandong province	empty stomach and guts, shell softening, muscle whitening and necrosis at the acute stage	+	+	+		+
181	20211228069	*P. vannamei*	Shandong province	empty stomach and guts, shell softening, muscle whitening and necrosis at the acute stage	+	+	+		+
182	20211228070	*P. vannamei*	Shandong province	empty stomach and guts, shell softening, muscle whitening and necrosis at the acute stage	+	+	+		+
183	20211228071	*P. vannamei*	Shandong province	empty stomach and guts, shell softening, muscle whitening and necrosis at the acute stage	+	+	+		+
184	20211228072	*P. vannamei*	Shandong province	empty stomach and guts, shell softening, muscle whitening and necrosis at the acute stage	+	+	+		+
185	20211228073	*P. vannamei*	Shandong province	empty stomach and guts, shell softening, muscle whitening and necrosis at the acute stage	+	+	+		+
186	20211228074	*P. vannamei*	Shandong province	empty stomach and guts, shell softening, muscle whitening and necrosis at the acute stage	+	+	+		+
187	20211228075	*P. vannamei*	Shandong province	empty stomach and guts, shell softening, muscle whitening and necrosis at the acute stage	+	+	+		+
188	20211228076	*P. vannamei*	Shandong province	empty stomach and guts, shell softening, muscle whitening and necrosis at the acute stage	+	+	+		+
189	20211228077	*P. vannamei*	Shandong province	empty stomach and guts, shell softening, muscle whitening and necrosis at the acute stage	+	+	+		+
190	20211228078	*P. vannamei*	Shandong province	empty stomach and guts, shell softening, muscle whitening and necrosis at the acute stage	+	+	+		+
191	20211228079	*P. vannamei*	Shandong province	empty stomach and guts, shell softening, muscle whitening and necrosis at the acute stage	+	+	+		+
192	20211228080	*P. vannamei*	Shandong province	empty stomach and guts, shell softening, muscle whitening and necrosis at the acute stage	+	+	+		+
193	20211228081	*P. vannamei*	Shandong province	empty stomach and guts, shell softening, muscle whitening and necrosis at the acute stage	+	+	+		+
194	20211228082	*P. vannamei*	Shandong province	empty stomach and guts, shell softening, muscle whitening and necrosis at the acute stage	+	+	+		+

195	20211228083	*P. vannamei*	Shandong province	empty stomach and guts, shell softening, muscle whitening and necrosis at the acute stage	+	+	+	+
196	20211228084	*P. vannamei*	Shandong province	empty stomach and guts, shell softening, muscle whitening and necrosis at the acute stage	+	+	+	+
197	20211228085	*P. vannamei*	Shandong province	empty stomach and guts, shell softening, muscle whitening and necrosis at the acute stage	+	+	+	+
198	20210917027	*Macrobrachium rosenbergii*	Jiangsu province	/	-	-	-	-
199	20210917028	*Macrobrachium rosenbergii*	Jiangsu province	/	-	-	-	-
200	20210917029	*Macrobrachium rosenbergii*	Jiangsu province	/	-	-	-	-
201	20210917030	*Macrobrachium rosenbergii*	Jiangsu province	/	-	-	-	-
202	20210917031	*Macrobrachium rosenbergii*	Jiangsu province	/	-	-	-	-
203	20210917032	*Macrobrachium rosenbergii*	Jiangsu province	/	-	-	-	-
204	20210917033	*Macrobrachium rosenbergii*	Jiangsu province	/	-	-	-	-
205	20210917034	*Macrobrachium rosenbergii*	Jiangsu province	/	-	-	-	-
206	20210917035	*Macrobrachium rosenbergii*	Jiangsu province	/	-	-	-	-
207	20210917036	*Macrobrachium rosenbergii*	Jiangsu province	/	-	-	-	-
208	20210917037	*Macrobrachium rosenbergii*	Jiangsu province	/	-	-	-	-
209	20210917038	*Macrobrachium rosenbergii*	Jiangsu province	/	-	-	-	-
210	20210917039	*Macrobrachium rosenbergii*	Jiangsu province	/	-	-	-	-
211	20210917040	*Macrobrachium rosenbergii*	Jiangsu province	/	-	-	-	-
212	20210917041	*Macrobrachium rosenbergii*	Jiangsu province	/	-	-	-	-
213	20210917042	*Macrobrachium rosenbergii*	Jiangsu province	/	-	-	-	-
214	20210917043	*Macrobrachium rosenbergii*	Jiangsu province	/	-	-	-	-
215	20210917044	*Macrobrachium rosenbergii*	Jiangsu province	/	-	-	-	-
216	20210917045	*Macrobrachium rosenbergii*	Jiangsu province	/	-	-	-	-
217	20210917046	*Macrobrachium rosenbergii*	Jiangsu province	/	-	-	-	-
218	20210917047	*Macrobrachium rosenbergii*	Jiangsu province	/	-	-	-	-
219	20210917048	*Macrobrachium rosenbergii*	Jiangsu province	/	-	-	-	-
220	20210917049	*Macrobrachium rosenbergii*	Jiangsu province	/	-	-	-	-
221	20210917050	*Macrobrachium rosenbergii*	Jiangsu province	/	-	-	-	-
222	20210917051	*Macrobrachium rosenbergii*	Jiangsu province	/	-	-	-	-
223	20210917052	*Macrobrachium rosenbergii*	Jiangsu province	/	-	-	-	-
224	20210917053	*Macrobrachium rosenbergii*	Jiangsu province	/	-	-	-	-
225	20210917054	*Macrobrachium rosenbergii*	Jiangsu province	/	-	-	-	-
226	20210917055	*Macrobrachium rosenbergii*	Jiangsu province	/	-	-	-	-
227	20210917056	*Macrobrachium rosenbergii*	Jiangsu province	/	-	-	-	-

228	20210917057	*Macrobrachium rosenbergii*	Jiangsu province	/	-	-	-	-
229	20210917058	*Macrobrachium rosenbergii*	Jiangsu province	/	-	-	-	-
230	20210917059	*Macrobrachium rosenbergii*	Jiangsu province	/	-	-	-	-
231	20210917060	*Macrobrachium rosenbergii*	Jiangsu province	/	-	-	-	-
232	20210917061	*Macrobrachium rosenbergii*	Jiangsu province	/	-	-	-	-
233	20210917062	*Macrobrachium rosenbergii*	Jiangsu province	/	-	-	-	-
234	20210917063	*Macrobrachium rosenbergii*	Jiangsu province	/	-	-	-	-
235	20210917064	*Macrobrachium rosenbergii*	Jiangsu province	/	-	-	-	-
236	20210917065	*Macrobrachium rosenbergii*	Jiangsu province	/	-	-	-	-
237	20210917066	*Macrobrachium rosenbergii*	Jiangsu province	/	-	-	-	-
238	20210917067	*Macrobrachium rosenbergii*	Jiangsu province	/	-	-	-	-
239	20210917068	*Macrobrachium rosenbergii*	Jiangsu province	/	-	-	-	-
240	20210917069	*Macrobrachium rosenbergii*	Jiangsu province	/	-	-	-	-
241	20210917070	*Macrobrachium rosenbergii*	Jiangsu province	/	-	-	-	-
242	20210917071	*Macrobrachium rosenbergii*	Jiangsu province	/	-	-	-	-
243	20210917072	*Macrobrachium rosenbergii*	Jiangsu province	/	-	-	-	-
244	20210917073	*Macrobrachium rosenbergii*	Jiangsu province	/	-	-	-	-
245	20210917074	*Macrobrachium rosenbergii*	Jiangsu province	/	-	-	-	-
246	20210917075	*Macrobrachium rosenbergii*	Jiangsu province	/	-	-	-	-
247	20210917076	*Macrobrachium rosenbergii*	Jiangsu province	/	-	-	-	-
248	20210917077	*Macrobrachium rosenbergii*	Jiangsu province	/	-	-	-	-
249	20210917078	*Macrobrachium rosenbergii*	Jiangsu province	/	-	-	-	-
250	20210917079	*Macrobrachium rosenbergii*	Jiangsu province	/	-	-	-	-
251	20210917080	*Macrobrachium rosenbergii*	Jiangsu province	/	-	-	-	-
252	20210917081	*Macrobrachium rosenbergii*	Jiangsu province	/	-	-	-	-
253	20210917082	*Macrobrachium rosenbergii*	Jiangsu province	/	-	-	-	-
254	20210917083	*Macrobrachium rosenbergii*	Jiangsu province	/	-	-	-	-
255	20210917084	*Macrobrachium rosenbergii*	Jiangsu province	/	-	-	-	-
256	20210917085	*Macrobrachium rosenbergii*	Jiangsu province	/	-	-	-	-
257	20210917086	*Macrobrachium rosenbergii*	Jiangsu province	/	-	-	-	-
258	20210917087	*Macrobrachium rosenbergii*	Jiangsu province	/	-	-	-	-
259	20210917088	*Macrobrachium rosenbergii*	Jiangsu province	/	-	-	-	-
260	20210917089	*Macrobrachium rosenbergii*	Jiangsu province	/	-	-	-	-
261	20210917090	*Macrobrachium rosenbergii*	Jiangsu province	/	-	-	-	-

262	20210917091	*Macrobrachium rosenbergii*	Jiangsu province	/	-	-	-	-
263	20210917092	*Macrobrachium rosenbergii*	Jiangsu province	/	-	-	-	-
264	20210917093	*Macrobrachium rosenbergii*	Jiangsu province	/	-	-	-	-
265	20210917094	*Macrobrachium rosenbergii*	Jiangsu province	/	-	-	-	-
266	20210917095	*Macrobrachium rosenbergii*	Jiangsu province	/	-	-	-	-
267	20210917096	*Macrobrachium rosenbergii*	Jiangsu province	/	-	-	-	-
268	20210917097	*Macrobrachium rosenbergii*	Jiangsu province	/	-	-	-	-
269	20210917098	*Macrobrachium rosenbergii*	Jiangsu province	/	-	-	-	-
270	20210917099	*Macrobrachium rosenbergii*	Jiangsu province	/	-	-	-	-
271	20210917100	*Macrobrachium rosenbergii*	Jiangsu province	/	-	-	-	-
272	20210917101	*Macrobrachium rosenbergii*	Jiangsu province	/	-	-	-	-
273	20210917102	*Macrobrachium rosenbergii*	Jiangsu province	/	-	-	-	-
274	20210917103	*Macrobrachium rosenbergii*	Jiangsu province	/	-	-	-	-
275	20210917104	*Macrobrachium rosenbergii*	Jiangsu province	/	-	-	-	-
276	20210917105	*Macrobrachium rosenbergii*	Jiangsu province	/	-	-	-	-
277	20210917106	*Macrobrachium rosenbergii*	Jiangsu province	/	-	-	-	-
278	20210917107	*Macrobrachium rosenbergii*	Jiangsu province	/	-	-	-	-
279	20210917108	*Macrobrachium rosenbergii*	Jiangsu province	/	-	-	-	-
280	20210917109	*Macrobrachium rosenbergii*	Jiangsu province	/	-	-	-	-
281	20210626001	*Macrobrachium rosenbergii*	Jiangsu province	/	+	+	+	-
282	20210626020	*Macrobrachium rosenbergii*	Jiangsu province	/	+	+	+	-
283	20210626041	*Macrobrachium rosenbergii*	Jiangsu province	/	+	-	+	-
284	20210626045	*Macrobrachium rosenbergii*	Jiangsu province	shell softening	+	+	+	-
285	20210626048	*Macrobrachium rosenbergii*	Jiangsu province	/	+	-	-	-
286	20210626050	*Macrobrachium rosenbergii*	Jiangsu province	shell softening, muscle whitening and necrosis at the acute stage	+	+	-	-
287	20210626051	*Macrobrachium rosenbergii*	Jiangsu province	/	+	+	-	-
288	20210626052	*Macrobrachium rosenbergii*	Jiangsu province	/	-	-	-	-
289	20210626053	*Macrobrachium rosenbergii*	Jiangsu province	/	+	+	-	-
290	20210626054	*Macrobrachium rosenbergii*	Jiangsu province	/	-	-	-	-
291	20210626055	*Macrobrachium rosenbergii*	Jiangsu province	/	+	-	-	-
292	20210626056	*Macrobrachium rosenbergii*	Jiangsu province	/	-	-	-	-
293	20210630001	*P. vannamei*	Shandong province	swimming ability decreased, shell softening, muscle whitening and necrosis at the acute stage	+	+	-	-
294	20210630002	*P. vannamei*	Shandong province	swimming ability decreased, shell softening, muscle whitening and necrosis at the acute stage	+	+	-	-

295	20210630003	*P. vannamei*	Shandong province	swimming ability decreased, shell softening, muscle whitening and necrosis at the acute stage	+	+	+	-
296	20210630012	*P. vannamei*	Shandong province	shell softening	-	-	-	-
297	20210917002	*Macrobrachium rosenbergii*	Jiangsu province	empty stomach and guts	-	-	-	-
298	20210917004	*Macrobrachium rosenbergii*	Jiangsu province	/	-	-	-	-
299	20210917005	*Macrobrachium rosenbergii*	Jiangsu province	empty stomach and guts, shell softening, muscle whitening and necrosis at the acute stage	+	+	+	-
300	20210917006	*Macrobrachium rosenbergii*	Jiangsu province	muscle whitening and necrosis at the acute stage	+	+	-	-

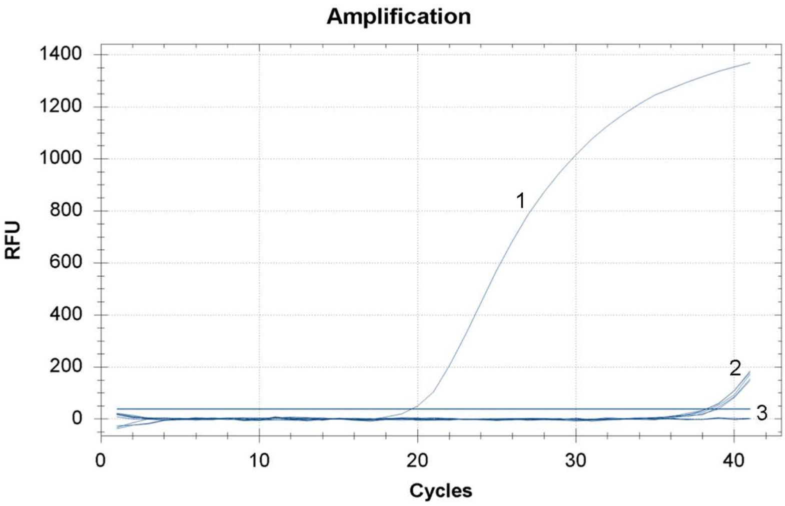

Supplemental Figure S1. The sensitivity detection results of the newly developed TaqMan RT-qPCR.

1. Positive control, 2. The total tissues RNA of healthy shrimp mixed 1.36 copies synthetic RNA standard, 3. Negative control.
